# The Fusion Application of Deep Learning Biological Image Visualization Technology and Human-Computer Interaction Intelligent Robot in Dance Movements

**DOI:** 10.1155/2022/2538896

**Published:** 2022-09-20

**Authors:** Nian Jin, Lan Wen, Kun Xie

**Affiliations:** ^1^College of Music and Dance, Guangzhou University, Guangzhou 510006, China; ^2^South China Business College, Guangdong University of Foreign Studies, Guangzhou 510545, China; ^3^Faculty of Education, Guangxi Normal University, Guilin 535400, China; ^4^Chongqing College of Humanities Science and Technology, Chongqing, China

## Abstract

The paper aims to apply the deep learning-based image visualization technology to extract, recognize, and analyze human skeleton movements and evaluate the effect of the deep learning-based human-computer interaction (HCI) system. Dance education is researched. Firstly, the Visual Geometry Group Network (VGGNet) is optimized using Convolutional Neural Network (CNN). Then, the VGGNet extracts the human skeleton movements in the OpenPose database. Secondly, the Long Short-Term Memory (LSTM) network is optimized and recognizes human skeleton movements. Finally, an HCI system for dance education is designed based on the extraction and recognition methods of human skeleton movements. Results demonstrate that the highest extraction accuracy is 96%, and the average recognition accuracy of different dance movements is stable. The effectiveness of the proposed model is verified. The recognition accuracy of the optimized F-Multiple LSTMs is increased to 88.9%, suitable for recognizing human skeleton movements. The dance education HCI system's interactive accuracy built by deep learning-based visualization technology reaches 92%; the overall response time is distributed between 5.1 s and 5.9 s. Hence, the proposed model has excellent instantaneity. Therefore, the deep learning-based image visualization technology has enormous potential in human movement recognition, and combining deep learning and HCI plays a significant role.

## 1. Introduction

Modern technologies, such as the Internet and multimedia technology, have developed rapidly. Multimedia systems based on computer information technology have been applied in many fields. The intelligent interactive multimedia is a new platform that develops under the foundation of computer technology [1, 2]. However, the applications of traditional multimedia systems are often independent and mechanized, which are inadequate to meet people's needs. Consequently, the human-computer interaction (HCI) technology emerged. People can interact with the multimedia engine and obtain the required media information quickly and efficiently via HCI. Besides, HCI technology can promote the accurate transmission of information and improve work efficiency [3–5], which has triggered a research boom. In daily life, people can directly express their thoughts or emotions through movements. Therefore, movement recognition and analysis have become a critical direction in the field of HCI and attracted widespread attention, which leads to the wide popularity of human movement-based recognition technology [6, 7].

With the advancement of social informatization, human beings have an increasing requirement in the intelligence level of computers. HCI no longer only depends on the original hardware-based interaction, and some relatively more intelligent interaction methods gradually appear in mass life. The face recognition, gesture recognition, and speech recognition systems constructed by machine learning technology have established a bridge between humans and computers [8]. The emergence of these convenient interaction modes has become a major development trend in the field of HCI. The development of HCI mode aims to enable the computer to serve and adapt to human needs well, so HCI focuses on humans instead of adapting to the computer. Therefore, the friendly interaction between robots and humans is extremely vital in the research of machine learning and HCI. Some scholars focus on the importance of emotional factors related to the interaction between people and computer systems, when exploring the people-centered interaction systems [9]. Motion recognition technology is essentially a classification problem close to machine learning [10].

The above research results imply that the development of the Internet and multimedia technology has made multimedia systems successfully applied to many fields. The friendly interaction between robots and human beings plays an extremely important role in the study of machine learning and HCI. Deep learning shows excellent application potential in function extraction and HCI. A combination of deep learning and HCI is innovatively proposed to extract and identify human skeleton operations to expand the application field of HCI. The ultimate research purpose is to achieve a significant reduction in time costs and dependence on traditional equipment and facilities. The innovative ideas can also achieve the purpose of improving human-computer collaboration and interaction. Moreover, combined with the image visualization technology based on deep learning and HCI system, it is envisaged that the visual geometric group network (VGGNet) and long short-term memory (LSTM) can be optimized. The final HCI system and the research results of the recognition and analysis of human dance provide a reference value.

The contributions based on the extraction and recognition of human dance movements are as follows:

1. An optimized VGGNet human skeleton movement extraction algorithm is proposed. Its extraction accuracy reaches 96%, which is significantly better than traditional algorithms.
2. An optimized multiple LSTM human skeleton movement recognition algorithm is proposed. Its recognition accuracy reaches 88.9%, which is significantly better than traditional LSTMs.
3. An HCI system based on image visualization is designed, and the interaction accuracy rate reaches 92%.
4. A reference is provided for more in-depth human movement extraction and recognition, and deep learning methods' application range in HCI systems is expanded.

## 2. Literature Review

### 2.1. Current Situation of Deep Learning in Dance Education

Dance is an important intangible cultural heritage. Dimitropoulos et al. introduced a research project (i-juries) of intangible cultural heritage, emphasizing the importance of 3D dance interaction [11]. Grammalidis et al. introduced an intangible cultural heritage dataset, i-treasure, including audio and other data information [12]. Doulamis et al. considered that intangible cultural heritage was an important source of cultural diversity, but there were few electronic documents of intangible cultural heritage. According to the “Terpsichore” project funded by the Horizon 2020 of the European Union, they proposed a high-level method based on the digitization of cultural assets [13]. Doulamis et al. discussed the digitization of tangible and intangible cultural heritage and proposed that 3D digital assets would develop into a part of augmented, virtual, and mixed reality experience [14]. Lv studied the application of virtual reality (VR) in 3D environment and HCI system and revealed the excellent performance of VR technology in 3D digitization [15]. The digitization of intangible cultural heritage has become an inevitable development trend, so has dance.

On the recognition and extraction of dance movements, Rallis et al. proposed a dance summarization method based on 3D capture data of the Vicon motion capture system. They analyzed and studied the automatic extraction of dance patterns. This method was a hierarchical scheme based on the temporal and spatial changes of dance characteristics [16]. Aiming at the preservation and dissemination of dance performance, Aristidou et al. proposed a dance action recognition framework based on Laban analysi which used feature space to capture different dance action components and pointed out a new direction for dance evaluation [17]. In terms of editing and synthesis of dance movements, Aristidou et al. used Laban analysis, radial basis function regression, and interpolation methods to map the movement features and emotional features in two directions and realized the stylization of high dynamic dance movements [18]. To sum up, there is a difference between the research of human action recognition and HCI, and there is little research on action recognition in dance education.

### 2.2. Research Progress of HCI

Experts and scholars have made great efforts on deep learning and HCI. Bhardwaj et al. applied support vector machine and artificial neural network classifier to fingerprint recognition. By integrating the relevant dynamic information from hundreds of biometric scanning sample datasets, they found that the accuracy of fingerprint dynamic recognition by fusing the deep learning method was improved by 5.3% [19]. Israelsen and Ahmed analyzed the influence of artificial intelligence (AI) agent in HCI and machine learning based on the research of algorithm-guaranteed AI agent and discussed the advantages and disadvantages of different methods [20]. Based on similarity embedding, Spathis et al. proposed an interactive dimension reduction framework (iSP). In this framework, user interaction formed different goals. Gradient descent was used for learning, and an end-to-end composition structure could be trained. By evaluating the framework in two interaction scenarios, they found that the framework could be applied to semisupervised learning, transfer learning, and adaptive learning in interaction field [21]. Using interactive machine learning, Wu et al. studied local decision-making in feature selection of emotion classification task and analyzed the influence of interactive machine learning tools on feature selection results [22]. To improve the performance of multimodal image retrieval by using unmarked and marked multimodal web objects, Xu et al. proposed a semisupervised multiconcept retrieval method based on deep learning (SMRDL). Different from the traditional method of using multiple independent concepts in multiconcept semantic query, the proposed method regarded multiple concepts as a whole scene, which was used for multiconcept scene learning of unimodal retrieval. The comprehensive experimental results on two datasets of MIR flickr2011 and NUS-WIDE indicated that the proposed method was superior to some of the latest methods [23]. Long and Zhao held that intelligent teaching mode overcame the shortcomings of traditional online and offline teaching. However, there were some shortcomings in the real-time feature extraction of teachers and students. In view of this, they used particle swarm image recognition and deep learning technology to process the video teaching image of intelligent classroom. To overcome the shortcomings of premature convergence of standard particle swarm optimization (PSO) algorithm, they proposed an improved multi PSO algorithm strategy. Moreover, to improve the premature problem of PSO in search performance, they combined the algorithm with the useful attributes of other algorithms to improve the diversity of particles in the algorithm, enhance the global search ability of particles, and achieve effective feature extraction [24]. To sum up, there are many research results on the application of deep learning in HCI, but few studies on the combination of the two for dance action extraction.

## 3. Methods

In computer vision and image processing, movement recognition is a crucial component. However, some problems are found in its research and applications. For example, when extracting and recognizing human skeleton movements, bone modeling is challenging, movement amplitude can affect the extraction results, and feature extraction can be insufficient, increasing the difficulty in analyzing and classifying human movements. Deep learning has developed rapidly. CNN shows excellent performance in feature extraction, while LSTM has significant performance in processing time sequence problems. Therefore, CNN and LSTM are introduced to extract and recognize human skeleton movements. However, traditional CNN models have lots of parameters, using a large convolution kernel to extract features. Traditional LSTM models never consider the connection of multiple different movement times in a long time. Hence, the CNN-based VGGNet is introduced and optimized in parallel. In the meantime, LSTM is improved and optimized before extracting and recognizing human skeleton movements.

### 3.1. Optimization of VGGNet CNN Model

Cat's visual cortex theory inspires the deep learning-based CNN. Compared with the traditional neural network, CNN extracts the object's local feature information through the convolution layer, a critical CNN component that contains multiple convolution kernels [25]. VGGNet is a typical CNN. Unlike traditional CNNs that employ big convolution kernels to extract features, VGGNet utilizes several 3*∗*3 small convolution kernels for feature extraction. Hence, VGGNet can extract richer features and reduce the calculation amount significantly [26–28].

The features extracted by the convolution layer are integrated to improve the accuracy of VGGNet, i.e., the parallel CNN [29–31].

Extractions of input image features before fusion are as follows:

(1) $$\begin{array}{c} y_{1}^{A}=F_{1}^{A}\left(x\right)\text{,} \end{array}$$

(2) $$\begin{array}{c} y_{1}^{B}=F_{1}^{B}\left(x\right)\text{.} \end{array}$$

In (1) and (2), *F*_1_^*A*^ and *F*_1_^*B*^ represent features. The feature information extracted by the two small convolution kernels is fused via the feature fusion module. The convolution operation is denoted as *G*. The feature map after fusion processing can be written as follows:

(3) $$\begin{array}{c} y_{1}^{c}=G_{1}\left(y_{1}^{A},y_{1}^{B}\right)=G_{1}\left(F_{1}^{A}\left(x\right),F_{1}^{B}\left(x\right)\right)\text{.} \end{array}$$

The process of fusion of the above feature maps *y*_1_^*c*^, *y*_1_^*A*^, and *y*_1_^*B*^ can be expressed as follows:

(4) $$\begin{array}{c} y_{1}^{c}=merge\left(y_{1}^{A},y_{1}^{B}\right)\text{.} \end{array}$$

The above fusion processing can enrich and diversify the extracted features. Graphics Processing Unit (GPU) processing is utilized for training VGGNet to compare the performance of the CNN-based VGGNet before and after optimization. Images in the training set are taken by the Kinect camera and the host computer program. The selected human movements include clapping, slapping, standing, picking up objects, and sitting down.

Movement capture includes the following steps: (1) the demonstrator makes different movements in front of the Kinect camera and (2) Kinect is utilized for evaluating human skeleton changes in real-time. Several demonstrators complete the collection of the entire training set. One thousand images are collected for each movement. Finally, a total of 5,000 human skeleton images under different movements are obtained. The skeleton images affected by the environment are removed, and the remaining human skeleton images are retained. These images train the VGGNet before and after feature fusion. Accuracy and loss rates are taken as evaluation indicators [32, 33]. Parameter settings of the entire training process are shown in Table 1.

### 3.2. Extraction Algorithm of Human Skeleton Movements

Traditional human pose estimation algorithms extract human skeleton features via the bottom-up manner. Each skeleton extraction object requires a detector, and each movement is estimated separately. Therefore, traditional algorithms have many problems, such as false detection, long-running time, and poor instantaneity, which cannot meet the demands. Based on the OpenPose open-source database [34], the optimized VGGNet is the network architecture, and the histogram equalization [35, 36] is introduced to suppress noises, thereby extracting the 2D features of the human skeleton.

**Figure 1:**
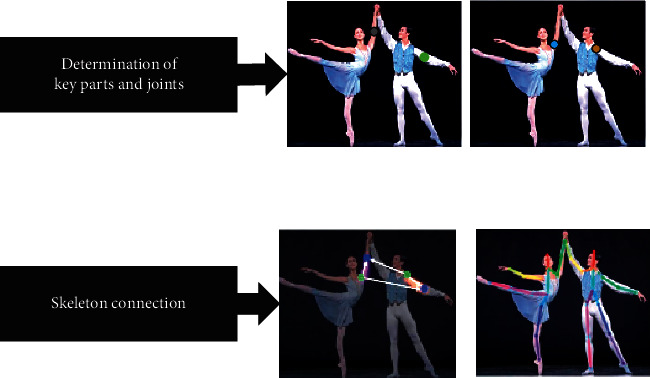
Video information extraction and processing based on open pose (Image URL: https://img-blog.csdnimg.cn/20200910162313905png?x-oss-processimage/watermark, type _ZmFuZ3poZW5naGVpdGk, shadow_10, text_aHR0cHM6Ly9ibG9nLmNzZG4ubmV0L3N1bm55YmxvZ3M, size_16, color_FFFFFF, t_70. Copyright statement URL: https://wwwcsdnnet/company/indexhtml#statement).

OpenPose is an open-source database released in 2017 based on skeleton extraction. Unlike traditional pose estimation algorithms, OpenPose uses a bottom-up method. The joint points of all human body parts are detected first. Then, the nodes are connected to obtain the skeleton, thereby significantly reducing the running time. Also, OpenPose can improve detection accuracy and shorten the running time.  Figure 1 illustrates the video information processing by OpenPose.

The unique convolution kernel structure in the CNN can learn spatial information in human actions, and more useful information can be obtained by different convolution kernels. Compared with traditional machine learning methods, CNN is more systematic and comprehensive in task learning with better performances. Unlike traditional CNN models, the VGGNet model extracts features by massive small convolution kernels as a typical CNN model. It can extract more features and reduce calculation amount with satisfactory generalization performance. The optimized VGGNet consists of three parts. The first part processes the image data via the input layer and employs CNN to extract the feature values of body parts. Then, the extracted feature values enter the other two parts for critical point positioning and the body-based 2D vector field positioning. The input to output via the neural network spends a total of *k* periods, and the information input to the current period is the output feature value obtained through the learning process of *k* − 1. The optimized VGGNet's output is formed by a 2D vector field of crucial body parts and a confidence map. As the calculations increase, the candidate human body parts and the corresponding structure division become apparent via this cyclic process. Here, CNN's first convolutional layer is a double convolutional layer, and each contains 64 convolution kernels in the size of 4*∗*4. Simultaneously, an activation layer and a normalization layer are added after each convolutional layer to process the nonlinear data. A pooling layer is added after the normalization layer to reduce dimensionality and prevent overfitting, located between the two convolutional layers. The Dropout layer comes after the second pooling layer. The Part Affinity Fields (PAFs) [37, 38] are adopted to predict all the human body key points in the images.

In summary, extracting human skeleton information includes the following two processes: first, adding the corresponding image data to the input layer of VGGNet and, second, learning the feature value *F* according to the body parts. The 2D vector field of output corresponding to the human body in the *k* *=* 1 period is

(5) $$\begin{array}{c} S^{t}=\rho^{t}\left(F,S^{t-1},L^{t-1}\right),\forall t\geq 2\text{,} \end{array}$$

(6) $$\begin{array}{c} L^{t}=\phi^{t}\left(F,S^{t-1},L^{t-1}\right),\forall t\geq 2\text{.} \end{array}$$

In (5) and (6), *S* represents the set of 2D position confidence maps, *ρ* and *ϕ* denote the set parameters, *t* refers to the period corresponding to the feature value, and *L* signifies the set of 2D vector fields.

The solution to the confidence in the confidence map can be presented as follows:

(7) $$\begin{array}{c} S_{j,k}^{*}\left(p\right)=\exp\;\left(-\frac{{\left\|p-x_{j,k}\right\|}_{2}^{2}}{\sigma^{2}}\right)\text{,} \end{array}$$

(8) $$\begin{array}{c} S_{j}^{*}\left(p\right)=\max_{k}S_{j,k}^{*}\left(p\right)\text{.} \end{array}$$

In (7) and (8), *S* represents the position confidence atlas and *p* denotes the output image in the corresponding period. Meanwhile, *k* refers to the number of people in the input image, *j* stands for the body part's serial number, and *σ* is a constant.

The joint point position in the 2D vector field is judged according to

(9) $$\begin{array}{c} L_{c,k}^{*}\left(p\right)=\left\{\begin{array}{c} v,\\ 0, \end{array}\right. \end{array}$$

(10) $$\begin{array}{c} v=\frac{\left(x_{j2,k}-x_{j1,k}\right)}{{\left\|x_{j2,k}-x_{j1,k}\right\|}_{2}}\text{.} \end{array}$$

In (9) and (10), *p* represents the pixel of the prejudgment part and *v* denotes the unit vector. On this basis, the average value of the 2D vector field can be written as follows:

(11) $$\begin{array}{c} L_{c}^{*}\left(p\right)=\frac{1}{n_{c\left(p\right)}}\underset{k}{\sum }L_{c.k}^{*}\left(p\right)\text{.} \end{array}$$

In (11), *n*_*c*(*p*)_ represents the number of all points of the pixel *p* on the link *c*. After testing, candidate positions on PAFs should be determined first. Then, all connected line segments are determined.

The OpenPose open-source library can achieve excellent results of skeleton extraction. However, the image noise limits feature extraction. Therefore, histogram equalization is introduced, which enhances the contrast and reduces the noise by stretching the distribution range of pixel intensity. Videos based on image visualization are processed by Compute Unified Device Architecture (CUDA) to ensure the instantaneity of information extraction. Eighteen key part points are chosen as the input of skeleton movement extraction while utilizing the OpenPose open-source library. A variable-view movement database containing 40 kinds of aerobic exercises is chosen for analyzing algorithm extraction effects. Eight different movements are chosen for analysis, with the classification accuracy as the primary evaluation indicator.

Here, the optimized 3D CNN (O-3DCNN) algorithm, Spatial-Temporal CNN(ST-CNN) algorithm, and optimized Deformable Part Model CNN (ODPM-CNN) are compared with the optimized VGGNet to prove its effectiveness.

### 3.3. Skeleton Movement Recognition Based on Optimized LSTM

Traditional neural networks have major limitations in practical application. For example, in time series processing, traditional methods perform well only in short-time series processing. In the separate data processing, the good learning and understanding abilities enable CNN to be applied in practice. However, CNN has limitations in the sequence problem processing related to time correlation. LSTM is a unique Recurrent Neural Network (RNN). LSTM can solve the long-term dependence problem in RNN applications, which has an inseparable relationship with the particular gate structure of LSTM, explicitly referring to input gates, forget gates, and output gates. The input data are calculated according to the following equation:

(12) $$\begin{array}{c} f_{t}=\sigma \left(w_{f}\left[h_{t-1},x_{t}\right]+b_{f}\right)\text{.} \end{array}$$

In (12), *w* represents the weight, *b* corresponds to the deviation, and *h*_*t*−1_ denotes the output value corresponding to the time *t* − 1. Meanwhile, *x*_*t*_ refers to the input value, *σ* represents the activation function, and *f* stands for the forget gate. Moreover, the memory information *c*_*t*_ can be displayed as follows:

(13) $$\begin{array}{c} c_{t}=f_{t}c_{t-1}+j_{t}\tanh\;\left(w_{c}\cdot \left[h_{t-1},x_{t}\right]+b_{c}\right)\text{.} \end{array}$$

In (13), *c*_*t*−1_ represents deciding whether to memorize the information at the time *t* − 1 and *j*_*t*_ means the input gate.

Finally, the output gate *o*_*t*_ can be expressed as follows:

(14) $$\begin{array}{c} o_{t}=\sigma \left(w_{o}\cdot \left[h_{t-1},x_{t}\right]+b_{o}\right)\text{.} \end{array}$$

**Figure 2:**
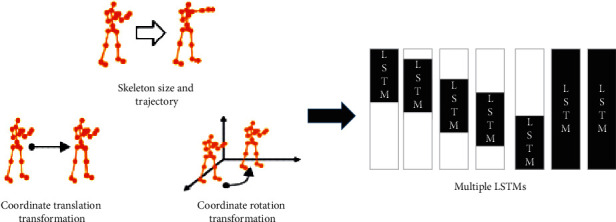
Overall implementation framework of multi-LSTM human skeleton movement recognition.

Although LSTM has many excellent performances, LSTM does not consider the correlation and feature influence between different skeleton movements over a long time. Hence, the LSTM model only depends on the human skeleton joints while recognizing human skeleton movements, resulting in limitations to recognizing human skeleton movements. Therefore, the idea of time integral is introduced. First, the pre-acquired skeleton sequence information is transformed, such as translation and rotation. In this way, all movements can obtain their relative coordinates. If the human skeleton movement has differences due to different times, a multiple LSTM model is used to extract and fuse features [39]. Finally, multiple types of movements are captured by integrating multiple LSTMs.  Figure 2 reveals the overall implementation framework of the optimized multi-LSTM human skeleton movement recognition.

Extraction accuracy and loss entropy of various LSTMs are compared to verify the effectiveness of the optimized multi-LSTM human skeleton movement recognition algorithm. Specifically, algorithms selected for comparison include the single-LSTM and double-LSTM. A skeleton sequence input into the optimized F-Multi-LSTM contains 24 frames, among which each frame consists of multiple 2D skeleton points. During analysis, the Adam optimization algorithm is used as the optimization tool, and the initial learning rate is set to 10^−4^, in an effort to achieve the model's global optimization. The single-LSTM has one input layer, while the double-LSTM has two input layers. The input is assumed as a sentence. In double-LSTM, one side of the input corresponds to the word at the beginning of the sentence and the other side corresponds to the word at the end of the sentence.

### 3.4. Design of HCI System Based on Dance Education

**Figure 3:**
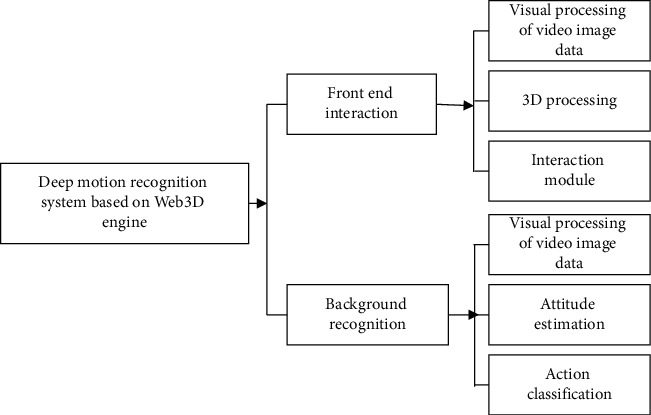
Functional modules of the deep motion recognition system.

Dance education based on physical education helps improve students' physical fitness and transforms traditional sports teaching. According to the above image visualization-based extraction and recognition method of human skeleton movements, the Web3D engine-oriented deep movement recognition system's functional modules are shown in Figure 3.

The system based on dance education and dance movement recognition consists of the front-end interactive function module and the back-end recognition function module. The former is a 3D world built on Web Graphics Library (WebGL) technology, including data processing of video images, 3D processing, and the HCI submodule. The latter consists of two subfunction modules, namely, node recognition and classification of human dance movements.

In this HCI system, the OpenPose open-source database and optimized VGGNet model can estimate facial expressions, positioning of limbs and trunk, and people's feature information. This human skeleton extraction method can identify the critical points of the human body, thereby employing the optimized F-Multi-LSTM skeleton movement recognition network to determine the classification and label attribution of human dance movements. The designed system is based on recognizing and analyzing dance movements. Eight types of dance movements are analyzed and discussed, including stepping and knee lift (*S*), crouching (*C*), reaching out and jumping (*R*), turning and clapping (*T*), straight punch (*B*), arm circles (*A*), jumping (*J*), and high knee (*H*).

In the HCI system, the dance pose estimation module and dance movement classification module in the background recognition module are the keys. Accuracy and response time are evaluation indicators to analyze the chosen dance movements, thereby testing the feasibility of the HCI system based on dance education and movement analysis and recognition.

### 3.5. Data Preprocessing

The image is preprocessed as follows to better meet the needs of behavior recognition: first, the image is uniformly scaled to 432 × 368 based on the center point; second, image denoising. Noises are common in images, in which Gaussian noise is the most common one. The Gaussian filter is used for processing to effectively suppress the Gaussian noise in the image. The one-dimensional Gaussian distribution and two-dimensional Gaussian distribution are shown in (15) and (16), respectively. The Gaussian filter function in open-source computer vision library (Open CV) is used to realize image denoising, and the relevant parameters are optimized.

(15) $$\begin{array}{c} G\left(x\right)=\frac{1}{\sqrt{2\pi }\sigma }e^{-\frac{x^{2}}{2\sigma^{2}}}\text{,} \end{array}$$

(16) $$\begin{array}{c} G\left(x,y\right)=\frac{1}{\sqrt{2\pi }\sigma^{2}}e^{-\frac{x^{2}+y^{2}}{2\sigma^{2}}}\text{.} \end{array}$$

## 4. Results

This section analyzes the optimized VGGNet algorithm's performance through comparison with several human skeleton movement extraction algorithms. The accuracy of the VGGNet algorithm in human skeleton movement extraction is analyzed and optimized on this basis. The effectiveness of the optimized model is verified. Besides, comparative analysis is conducted on the performance of the LSTM model, the single-LSTM model, and the double-LSTM model. Finally, the interaction accuracy and system real-time performance shall prevail to verify the HCI dance education system's performance.

### 4.1. Performance Comparison of Skeleton Movement Extraction Algorithms

Table 2 presents the comparison result of the extraction accuracy of human movements by several algorithms, including the original and optimized VGGNet.

Table 2 suggests that the optimized VGGNet algorithm presents the best performance in extracting human movements, with the highest accuracy of 98.2%, showing apparent superiority in performance over traditional VGGNet algorithms. The 3D CNN model can only extract a type of features from a three-dimensional space because the weights of the convolution kernel are the same in the whole space; that is, the weights are shared by the same convolution kernel, so the extraction accuracy of 3D CNN is only 91.2%. The spatial invariance of ST-CNN refers to the invariance of spatial transformation of images such as rotation, translation, and scaling. Even if the input is transformed or slightly modified, the model can recognize and extract features. ST-CNN is the most time-consuming and error-prone place in debugging interpolation and image index, so the extraction accuracy of ST-CNN is only 90.5%. ODPM-CNN model is a variability network and ODPM-CNN just the opposite, and its recognition accuracy reached 97.08%. The optimized VGGNet is also superior to other human movement extraction algorithms. In this way, the effectiveness of the proposed skeleton extraction algorithm is verified preliminarily.

### 4.2. Extraction Results of Human Skeleton Movements

**Figure 4:**
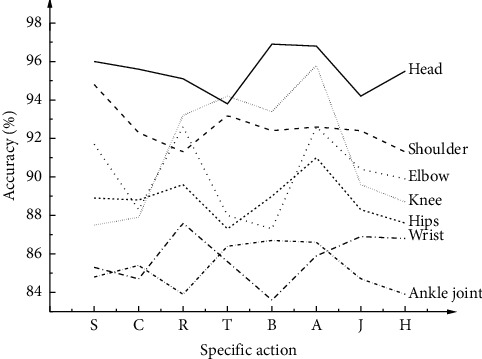
Accuracy distribution of movement extraction results of the human skeleton. (Labels on the *x*-axis represent dance movements. *S* represents stepping and knee lift, *C* represents crouching, *R* stands for reaching out and jumping, *T* stands for turning and clapping, *B* denotes straight punch, *A* denotes arm circles, *J* refers to jumping, and *H* refers to high knee).

The accuracy distribution of the eight human skeleton movements' extraction results by optimized VGGNet on OpenPose open-source database is shown in Figure 4.

This collection of 100 dance pictures is seen as a total sample, and each picture contains eight parts of the action changes. *S* represents the step and knee lifting head, shoulders, elbows, wrists, hips, knees, ankle bone node extraction accuracy; other *C*, *R*, *T*, *B*, *A*, *J*, and *H* dataset content for the above eight parts of the extraction accuracy changes under the action of the title annotation. The extraction accuracy of the head is the highest, reaching 96%, and 100 images are correctly extracted. The extraction accuracy of the shoulder reaches 84.8%, with 90 pictures extracted correctly. The extraction accuracy of the elbow reaches 92.6%, with 89 pictures extracted correctly. The extraction accuracy of the wrist reaches 87.6%, with 86 pictures correctly extracted. The extraction accuracy of the hip reaches 91.0%, with 100 pictures extracted correctly. The extraction accuracy of the knee reaches 95.8%, with 90 pictures extracted correctly. The extraction accuracy of the ankle reaches 86.7%, with 88 pictures extracted correctly. Figure 4 signifies that the extraction accuracy of bone nodes in eight body parts is different, and the proportion of sample number is also different. Moreover, Figure 4 implies that the proportion of accurate number extracted from the large part of the space occupied by the body parts will be significantly higher.

### 4.3. Skeleton Movement Recognition Results of Multiple LSTMs

**Figure 5:**
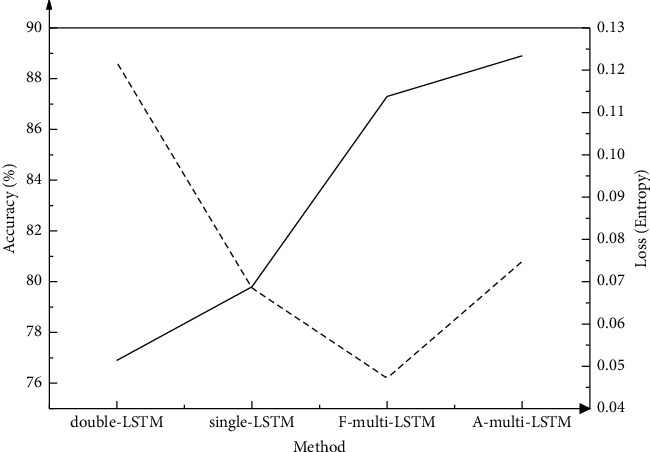
Comparison results of LSTM models.

The single-LSTM, double-LSTM, F-multi-LSTM, and A-multi-LSTM are compared. The results are shown in Figure 5.

The parameters represented by the abscissa in Figure 5 are different neural network models. The corresponding left-axis variables refer to the accuracy, and the corresponding right-axis variables stand for loss rates. Single-LSTM is a sequence that supports one-way variable input and output, while double-LSTM is a sequence that supports two-way input and output. Multi-LSTM is a multidimensional LSTM for high-frequency time series, which supports multiple parallel input sequences with multiple inputs, rather than the planar structure of multiple inputs in other models. F-Multi-LSTM is an optimized multidimensional LSTM, and A-Multi-LSTM is expressed as a pair of optimized multidimensional LSTM. The double-LSTM has higher accuracy than the single-LSTM according to the comparison results of loss rate and accuracy of single-LSTM and multi-LSTM. The recognition accuracy reaches 79.8%, and the loss rate is 0.0685. Compared with the single-LSTM model, the difference is 43.8%; overall, the recognition accuracy and loss rate of the proposed multi-LSTM model are the best. Specifically, the single-LSTM model's recognition accuracy reaches 88.9%, and the loss rate is 0.0748, which is the best among the comparative algorithms. Compared with the traditional LSTM model before improvement, the optimized LSTM model has higher recognition accuracy. The optimized LSTM model has the best applicability in recognizing human skeleton movements.

### 4.4. HCI System Performance Based on Dance Education and Movement Analysis

**Figure 6:**
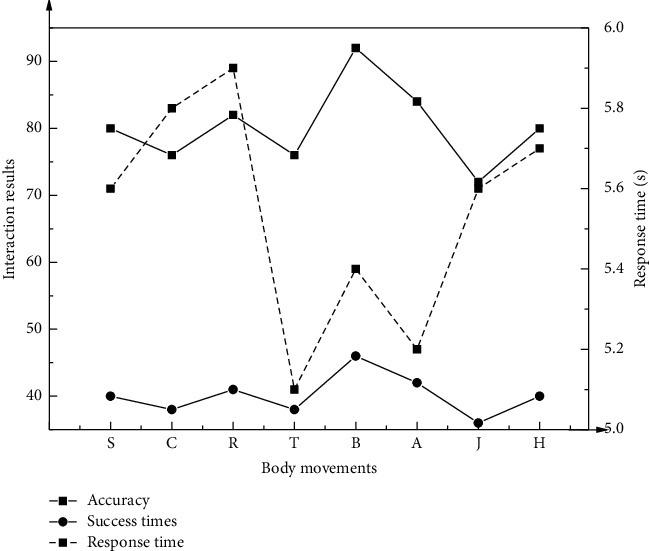
HCI system performance in terms of accuracy and instantaneity.

The eight dance movements are chosen as the benchmark. According to the indicators of interaction accuracy and system instantaneity, the HCI system's performance for dance education is shown in Figure 6.

In the dance education HCI system, the eight dance movements' overall interaction accuracy is above 70%. The interaction accuracy of movement B is the highest, reaching 92%. The overall accuracy of interactive recognition is distributed in the range of 72%–92%, with a large span. The overall response time corresponding to the eight dance movements is distributed within 5.1 seconds to 5.9 seconds, showing that the dance education HCI system has a high instantaneity.

## 5. Discussion

The above results indicate changes in the OpenPose open-source database's recognition accuracy and the optimized VGGNet model. The reason is that the head has almost no changes in coordinates or rotation angle. Besides, the movement range of the head is small. Therefore, the accuracy of classification and recognition of the head is the highest. In contrast, the shoulders are greatly affected by external factors, such as rotation angle and abscissa among different movements. Hence, classification and recognition accuracy of the shoulders are relatively low. The elbow movements and the wrist movements are affected by changes in moving speed and longitudinal coordinates. If the human body's moving speed is slow and the position between the arm and the camera is not parallel, the classification and recognition accuracy will be high. The hips are easily affected by changes in the leg movements. The overall accuracy of classification and recognition corresponding to the knees is high, but movements with large fluctuations, such as movement *H*, can significantly affect classification and recognition accuracy. Therefore, the accuracy is low. The ankles and other parts' classification and recognition accuracies are low, probably because of external factors such as clothes and shoes. Although the classification and recognition accuracy of different dance movements are mainly different, the average accuracy is high, confirming the proposed algorithm's effectiveness.

The multiple LSTM model is also advantageous in skeleton movement recognition. Because the optimized LSTM model is robust, its learning and classification abilities are increased, thereby increasing its accuracy in recognizing different dance movements. The distribution changes of the interaction accuracy corresponding to the dance education HCI system reveal that the interaction accuracy corresponding to different movements has a large span. Compared with the model training process, the actual interaction will be affected by sophisticated environmental conditions, such as different lighting, the restraint between different dance movements, and the conversion frequency of various dance movements. Under sophisticated environmental conditions, the interaction accuracy of the HCI system drops. Hence, attention should also be paid to improve datasets in actual HCI applications.

Meanwhile, the proposed algorithm is compared with the methods proposed by other scholars [40–49] to verify its superiority. For the training, the input image size is set to 432 × 368, the number of cycles is set to 50, the batch size is set to 16, and the initial learning rate is set to 0.001.  Table 3 reflects the results.  Table 3 demonstrates that the proposed multi-LSTM model has the highest accuracy in bone motion recognition, and the recognition accuracy has been improved by 27.79%, 17.69%, and 27.62%, respectively, compared with the comparative methods.

## 6. Conclusions

For the dance education HCI system, the CNN-based VGGNet model is optimized and applied to extract human skeleton movements based on the OpenPose open-source database and histogram equalization. The proposed extraction algorithm for human skeleton movements shows intentional performance in extracting eight different dance movements, with the highest accuracy rate reaching 96%. From the comparison results of loss value and accuracy between a single-LSTM model and a multi-LSTM model, the accuracy of bone motion recognition by the multi-LSTM model is 79.8%, which is higher than that by a single-LSTM model. The optimized multi-LSTM model has higher accuracy in recognizing human skeleton movements than the traditional LSTM models. The constructed HCI system has an interaction accuracy of 92%. This work achieves the extension of application range of deep learning in skeleton movement recognition and the organic combination of deep learning and HCI.

The contributions based on the extraction and recognition of human dance movements are as follows:

1. An optimized VGGNet human skeleton movement extraction algorithm is proposed, which achieves a better extraction accuracy than traditional algorithms, attaining 96%.
2. An optimized multiple LSTM human skeleton movement recognition algorithm is proposed. Its recognition accuracy reaches 88.9%, which is significantly better than traditional LSTMs.
3. A HCI system based on image visualization is designed, with the interaction accuracy rate of 92%.
4. A reference is provided for more in-depth human movement extraction and recognition, and deep learning strengthens the applicability to the HCI system.

Due to computational resource limitations, other larger and more complex datasets are considered in this experiment [50–55]. In addition, the algorithm can meet the real-time requirements, the recognition speed is still very slow. In view of the above problems, it is worth further expanding the datasets in complex scenes in the subsequent work and further optimizing the model to improve the detection speed [56].

Limited by the computing resources, other larger and more complex datasets are not explored [57, 58]. In addition, the recognition speed of the algorithm is slow although it can meet the real-time performance [59–61]. In view of the above problems, the dataset will be further expanded, especially in complex scenarios, which further optimizes the model to improve the speed of detection.

## Figures and Tables

**Table 1:** VGGNet training parameter settings.

Parameters	Training times	Learning rate	Number of images read	Optimizer
Corresponding value	500	10^−5^	32	Adam

**Table 2:** Comparison of several algorithms' extraction accuracy.

Algorithms	Original VGGNet	Optimized VGGNet	O-3DCNN	ST-CNN	ODPM-CNN
Extraction accuracy (%)	96.9	98.2	91.2	90.5	97.08

**Table 3:** Comparison of experimental results of different models.

Algorithms	Accuracy (%)
Method 1 [40]	61.11
Method 2 [41]	71.21
Method 3 [42]	61.28
Multi-LSTM model	88.9

## Data Availability

The raw data supporting the conclusions of this article will be made available by the authors, without undue reservation.

## References

[1] Kohek S., Strnad D., Zalik B., Kolmanic S. (2019). Interactive synthesis and visualization of self-organizing trees for large-scale forest succession simulation. *Multimedia Systems*.

[2] Canovas A., Jimenez J. M., Romero O., Lloret J. (2018). Multimedia data flow traffic classification using intelligent models based on traffic patterns. *IEEE Network*.

[3] Rapp A. (2020). Design fictions for learning: a method for supporting students in reflecting on technology in Human-Computer Interaction courses. *Computers & Education*.

[4] Hibbeln M., Jenkins J. L., Schneider C., Valacich J. S., Weinmann M. (2017). How is your user feeling? Inferring emotion through human-computer interaction devices. *MIS Quarterly*.

[5] Bergstrm J., Hornbk K. (2019). Human--Computer interaction on the skin. *ACM Computing Surveys*.

[6] Malgireddy M. R., Nwogu I., Govindaraju V. (2017). Language-motivated approaches to action recognition. *Journal of Machine Learning Research*.

[7] Singh S., Arora C., Jawahar C. V. (2017). Trajectory aligned features for first person action recognition. *Pattern Recognition*.

[8] Kim D. J., Song W. K., Han J. S., Bien Z. Z. (2003). Soft computing based intention reading techniques as a means of human-robot interaction for human centered system. *Soft Computing - A Fusion of Foundations, Methodologies and Applications*.

[9] Mano L. Y., Faiçal B. S., Gonçalves V. P. (2020). An intelligent and generic approach for detecting human emotions: a case study with facial expressions. *Soft Computing*.

[10] Erkan U. (2020). A Precise and Stable Machine Learning Algorithm: Eigenvalue Classification (EigenClass). *Neural Computing and Applications*.

[11] Dimitropoulos K., Manitsaris S., Tsalakanidou F., Spiros N Capturing the Intangible: An Introduction to the I-Treasures Project.

[12] Grammalidis N., Dimitropoulos K., Tsalakanidou F., Kitsikidis A The I-Treasures Intangible Cultural Heritage Dataset.

[13] Doulamis A., Voulodimos A., Doulamis N., Soile S Transforming Intangible Folkloric Performing Arts into Tangible Choreographic Digital Objects: The Terpsichore Approach.

[14] Doulamis N., Doulamis A., Ioannidis C., Klein M (2017). Modelling of Static and Moving Objects: Digitizing Tangible and Intangible Cultural Heritage. *Mixed Reality and Gamification for Cultural Heritage*.

[15] Lv Z. (2019). Virtual reality in the context of Internet of things. *Neural Computing & Applications*.

[16] Rallis I., Doulamis N., Doulamis A., Voulodimos A., Vescoukis V. (2018). Spatio-temporal summarization of dance choreographies. *Computers & Graphics*.

[17] Aristidou A., Stavrakis E., Charalambous P., Chrysanthou Y., Himona S. L. (2015). Folk dance evaluation using laban movement analysis. *Journal on Computing and Cultural Heritage*.

[18] Aristidou A., Zeng Q., Stavrakis E., Yin K Emotion control of unstructured dance movements.

[19] Bhardwaj I., Londhe N. D., Kopparapu S. K. (2019). Performance evaluation of fingerprint dynamics in machine learning and score level fusion framework. *IETE Technical Review*.

[20] Israelsen B. W., Ahmed N. R. (2019). Dave. I can assure you. That it’s going to be all right A definition, case for, and survey of algorithmic assurances in human-autonomy trust relationships. *ACM Computing Surveys*.

[21] Spathis D., Passalis N., Tefas A. (2019). Interactive dimensionality reduction using similarity projections. *Knowledge-Based Systems*.

[22] Wu T., Weld D. S., Heer J. (2019). Local decision pitfalls in interactive machine learning: an investigation into feature selection in sentiment analysis. *ACM Transactions on Computer-Human Interaction*.

[23] Xu H., Huang C., Wang D. (2019). Enhancing semantic image retrieval with limited labeled examples via deep learning. *Knowledge-Based Systems*.

[24] Long S., Zhao X. (2020). Smart teaching mode based on particle swarm image recognition and human-computer interaction deep learning. *Journal of Intelligent and Fuzzy Systems*.

[25] Zhang F., Cai N., Wu J., Cen G., Wang H., Chen X. (2018). Image denoising method based on a deep convolution neural network. *IET Image Processing*.

[26] Singh D., Erinc M, Sten H, Johannes K, Holzinger A., Goebel R., Ferri M., Palade V. (2017). Convolutional and recurrent neural networks for activity recognition in smart environment. *Towards Integrative Machine Learning and Knowledge Extraction: BIRS Workshop, Banff, AB, Canada, July 24-26, 2015, Revised Selected Papers*.

[27] Wong C. C., Gan Y., Vong C. M. (2020). Efficient outdoor video semantic segmentation using feedback-based fully convolution neural network. *IEEE Transactions on Industrial Informatics*.

[28] Tahir M., Tayara H., Chong K. T. (2019). iRNA-PseKNC(2methyl): identify RNA 2’-O-methylation sites by convolution neural network and Chou’s pseudo components. *Journal of Theoretical Biology*.

[29] Yao Q., Wang R., Fan X., Liu J., Li Y. (2020). Multi-class Arrhythmia detection from 12-lead varied-length ECG using attention-based time-incremental convolutional neural network. *Information Fusion*.

[30] Cheng P. M., Malhi H. S. (2017). Transfer learning with convolutional neural networks for classification of abdominal ultrasound images. *Journal of Digital Imaging*.

[31] Ardakani A., Condo C., Gross W. J. (2020). Fast and efficient convolutional accelerator for edge computing. *IEEE Transactions on Computers*.

[32] Hammad M., Wang K. (2019). Parallel score fusion of ECG and fingerprint for human authentication based on convolution neural network. *Computers & Security*.

[33] Yao P., Wu H., Gao B. (2020). Fully hardware-implemented memristor convolutional neural network. *Nature*.

[34] Dong Z., Du X., Liu Y. (2020). Automatic segmentation of left ventricle using parallel end-end deep convolutional neural networks framework. *Knowledge-Based Systems*.

[35] Yasoubi A., Hojabr R., Modarressi M. (2017). Power-efficient accelerator design for neural networks using computation reuse. *IEEE Computer Architecture Letters*.

[36] Sellami A., Hwang H. (2019). A robust deep convolutional neural network with batch-weighted loss for heartbeat classification. *Expert Systems with Applications*.

[37] Holzinger A., Goebel R., Ferri M. (2017). Towards integrative machine learning and knowledge extraction: BIRS workshop, banff, AB, Canada, july 24-26, 2015, revised selected papers. *Lecture Notes in Computer Science*.

[38] Mueggler E., Rebecq H., Gallego G., Delbruck T., Scaramuzza D. (2017). The event-camera dataset and simulator: event-based data for pose estimation, visual odometry, and SLAM. *The International Journal of Robotics Research*.

[39] Shakeri M., Dezfoulian M. H., Khotanlou H., Barati A., Masoumi Y. (2017). Image contrast enhancement using fuzzy clustering with adaptive cluster parameter and sub-histogram equalization. *Digital Signal Processing*.

[40] Shi Y., Wei Y., Pan D. (2019). Student body gesture recognition based on Fisher broad learning system. *International Journal of Wavelets, Multiresolution and Information Processing*.

[41] Huang W., Li N., Qiu Z. ., J., Jiang N., Wu B., Liu B. (2020). An automatic recognition method for students’ classroom behaviors based on image processing. *Traitement du Signal*.

[42] Cheng Y. Y., Dai Z. J., Ji Y., Simin L Student action recognition based on deep convolutional generative adversarial network.

[43] Memiş S., Enginoğlu S., Erkan U. (2021). Numerical data classification via distance-based similarity measures of fuzzy parameterized fuzzy soft matrices. *IEEE Access*.

[44] Liu R., Wang X., Lu H. (2021). SCCGAN: style and characters inpainting based on CGAN. *Mobile Networks and Applications*.

[45] Zhou W., Liu J., Lei J., Yu L., Hwang J. N. (2021). GMNet: graded-feature multilabel-learning network for RGB-thermal urban scene semantic segmentation. *IEEE Transactions on Image Processing*.

[46] Sun G., Cong Y., Wang Q., Zhong B., Fu Y. (2020). Representative task self-selection for flexible clustered lifelong learning. *IEEE Transactions on Neural Networks and Learning Systems*.

[47] Liu F., Zhang G., Lu J. (2021). Multisource heterogeneous unsupervised domain adaptation via fuzzy relation neural networks. *IEEE Transactions on Fuzzy Systems*.

[48] Yang W., Chen X., Xiong Z., Xu Z., Liu G., Zhang X. (2021). A privacy-preserving aggregation scheme based on negative survey for vehicle fuel consumption data. *Information sciences*.

[49] Li D., Ge S. S., Lee T. H. (2022). Simultaneous arrival to origin convergence: sliding-mode control through the norm-normalized sign function. *IEEE Transactions on Automatic Control*.

[50] Zhu B., Zhong Q., Chen Y. (2022). A novel reconstruction method for temperature distribution measurement based on ultrasonic tomography. *IEEE Transactions on Ultrasonics, Ferroelectrics, and Frequency Control*.

[51] Zheng W., Tian X., Yang B. (2022). A few shot classification methods based on multiscale relational networks. *Applied Sciences*.

[52] Wu X., Zheng W., Chen X., Zhao Y., Yu T., Mu D. (2021). Improving high-impact bug report prediction with combination of interactive machine learning and active learning. *Information and Software Technology*.

[53] Wang Y., Wang H., Zhou B., Fu H. (2021). Multi-dimensional prediction method based on Bi-LSTMC for ship roll. *Ocean Engineering*.

[54] Ban Y., Liu M., Wu P. (2022). Depth estimation method for monocular camera defocus images in microscopic scenes. *Electronics*.

[55] Zheng W., Liu X., Yin L. (2021). Research on image classification method based on improved multi-scale relational network. *PeerJ Computer Science*.

[56] Wang J., Tian J., Zhang X. (2022). Control of time delay force feedback teleoperation system with finite time convergence. *Frontiers in Neurorobotics*.

[57] Li J., Xu K., Chaudhuri S., Yumer E., Zhang H., Guibas L. (2017). Grass: generative recursive autoencoders for shape structures. *ACM Transactions on Graphics*.

[58] Qi M., Cui S., Chang X. (2022). Multi-region Nonuniform Brightness Correction Algorithm Based on L-Channel Gamma Transform. *Security and Communication Networks*.

[59] Zheng W., Yin L. (2022). Characterization inference based on joint-optimization of multi-layer semantics and deep fusion matching network. *PeerJ Computer Science*.

[60] Wang W., Chen Z., Yuan X. (2022). Simple low-light image enhancement based on Weber-Fechner law in logarithmic space. *Signal Processing: Image Communication*.

[61] Zhou W., Yu L., Zhou Y., Qiu W., Wu M. W., Luo T. (2018). Local and global feature learning for blind quality evaluation of screen content and natural scene images. *IEEE Transactions on Image Processing*.

